# Does a peer review group consensus process for MR-Linac patients affect clinical care? Evaluation of impact and feasibility

**DOI:** 10.1016/j.ctro.2024.100816

**Published:** 2024-07-03

**Authors:** Yew Sin, Vikneswary Batumalai, Jeremy de Leon, Eugene Leong, Kasri Rahim, Farshad Kasraei, Charles Tran, Tommy Liang, Katrina Biggerstaff, Michael G. Jameson, Nicole Hug, Kathryn Hird, Hendrick Tan

**Affiliations:** a University of Notre Dame Australia, School of Medicine, Fremantle, Western Australia, Australia; b GenesisCare, St Vincent’s Hospital, New South Wales, Australia; c The George Institute for Global Health, Faculty of Medicine and Health, UNSW Sydney, New South Wales, Australia; d GenesisCare, Murdoch, Western Australia, Australia; e Fiona Stanley Hospital, Department of Radiation Oncology, Murdoch, Western Australia, Australia; f The University of New South Wales, Sydney, Australia

**Keywords:** Radiation Oncology, Radiotherapy, Peer Review, Quality assurance, MR-linac, MR-guided radiotherapy, Adaptive radiotherapy, Stereotactic ablative body radiation therapy, Reirradiation

## Abstract

**Background and purpose:**

Peer review is an important component of quality assurance in radiotherapy. To our knowledge, there are no studies reporting on the feasibility and outcomes of the peer review process for magnetic resonance (MR) guided radiotherapy (MRgRT) on the MR linear accelerator (MR-Linac) despite the planning complexity involved and its evolving clinical indications. This study aimed to quantify the rate of change in treatment plans post-peer review and the time and resources required.

**Materials and methods:**

Fifty-five cases presented at weekly MR-Linac peer review meetings across two centres from 8 June to 21 September 2023 were prospectively collected. Cases were analysed to determine the rate and extent of plan changes based on the Peer Review Audit Tool for radiation oncology (PRAT) developed by the Royal Australian and New Zealand College of Radiologists (RANZCR).

**Results:**

Peer review resulted in changes to 36.4 % of treatment plans (n = 20), with 3.6 % (n = 2) having major changes requiring deferment of treatment. The most frequent changes were to organs at risk (OAR) volumes involving both delineation and increased OAR sparing (16.4 %, n = 9), total dose and fractionation (10.9 %, n = 6) and target volume dose coverage (5.5 %, n = 3). Patients with SBRT plans (39.1 % cf 22.2 %), oligometastatic/oligoprogressive sites (38.1 % cf 30.7 %) and reirradiation cases (41.2 % cf 34.2 %) had higher rates of change. Cases took a mean of 7 min (range 2–15 minutes) to discuss.

**Conclusion:**

The high rates of plan changes support the value of peer review in MRgRT. We recommend, where possible that all MRgRT cases, particularly those involving SBRT plans, oligometastatic/oligoprogressive sites, and/or reirradiation, be subject to peer review.

## Highlights

• Two-centre review of weekly MR-Linac peer review using RANZCR PRAT.
• Peer review resulted in considerable changes to MR-Linac treatment plans.
• SBRT plans and retreatment cases had higher rates of change after peer review.
• MRgRT cases, especially SBRT or reirradiation cases, should be subject to peer review.

## Introduction

Peer review conducted by a multidisciplinary team is an essential component of quality assurance in radiotherapy, given the multistep process involved in treatment planning. This complex planning process incorporates the subjectivity of clinical decision-making and is susceptible to inherent systemic errors, including human errors [1]. Barriers to effective peer review, such as workload, protected time, scheduling, attendance by key personnel, and culture, further underscore the challenges in maintaining rigorous quality assurance [1], [2].

The Royal College of Radiologists defines peer review as formal review by another site-specific oncologist of delineated contours. This robust review process, together with appropriate quality assurance processes, should be applied to contour delineation for organs at risk (OAR), especially for OARs that are more difficult to define [3] or in areas with variable physiological filling like luminal organs, which contribute to setup uncertainties and changes in internal anatomy [4]. Accordingly, the Royal Australian and New Zealand College of Radiologists (RANZCR) introduced the peer review audit tool (PRAT) as a quality improvement instrument [5]. The PRAT entails selecting patients for audit, conducting a preliminary scoring of each patient based on audit criteria by reviewing relevant patient documentation, conducting peer review of radiotherapy volumes and plans and re-auditing cases where a change in management was proposed.

The emergence of the magnetic resonance linear accelerator (MR-Linac) introduces a new treatment paradigm, offering improved visualisation of targets and OARs, coupled with the ability to adapt treatment plans in real time. While trials like ADAPT-MRL [6] and the MOMENTUM study [7] assess the technical feasibility and clinical outcomes of MR-Linac, there is a notable lack of studies reporting on the feasibility and effects of peer review processes specific to MR-Linac. Peer review is arguably essential for the MR-Linac treatment process, given the importance of accurate contouring and dosing to its clinical indications, especially in the context of stereotactic body radiotherapy (SBRT). Peer review also allows discussion of adaptive aspects of MR-Linac treatment planning, such as contouring for day-to-day variations in tumour size, shape and location, and structures with different visibility across CT and MRI (e.g. stents). In addition, some MR-Linac clinical indications have specific aspects of treatment planning that require significant emphasis in the review process. For example, pancreatic cancer cases require more focus on contour delineation due to the adjacency of the pancreas to sensitive OARs and the high doses of ablative radiation involved [4], [8]. Our practice has developed a group consensus peer review model based on the PRAT, involving a multidisciplinary review of treatment plans prior to the first fraction of radiation treatment. As peer review has been standard practice for non-MRgRT patients at our centres, it was introduced for MRgRT patients to ensure appropriate patient and indication selection, in addition to allowing us to identify any potential technical issues.

In this study we present the extent to which a prospective group consensus peer review process changed MR-Linac treatment plans and the amount of time and resources required for peer review to create a model of group consensus peer review that could be implemented in other MR-Linac departments.

## Materials and methods

Cases were presented at weekly MR-Linac peer review meetings across two cancer therapy centres in New South Wales and Western Australia in Australia, and prospectively collected over 16 weeks. These cases were reviewed to quantify the rate and extent of plan changes after the meetings. Both centres are equipped with the Unity MR-Linac (Elekta AB, Stockholm, Sweden), which allows the creation of reference plans and adaption to position and shape for daily plan modification [9]. Both centres maintain uniformity in simulation, planning and treatment protocols, including dose prescription, motion management and MR scanning, across all tumour sites. One centre started MRgRT in 2020 and provided support to the second centre which started MRgRT in 2022. Following the standard protocol, all patients underwent CT and MR simulation. The target volumes and OARs were delineated on planning CT by the radiation oncologist during the reference planning stage with the assistance of MR imaging, and MR imaging was used for target delineation for adapted fractions. For treatment planning, images were acquired on the MR-Linac at a simulation session based on each subsite’s exam cards, including T1, T2, T2 navigation, 3D Vane, diffusion-weighted imaging (DWI) and BTFE motion scans, with the treating radiation oncologist determining the MR sequence of choice (including contrast scans as required) for contouring both targets and OARs online [10]. The radiation therapists proceeded with treatment planning, and the final plan was reviewed by the radiation oncologist before presentation at the peer review meeting. To ensure a comprehensive review, all cases deemed complex (i.e., SBRT, reirradiation, or rare cases as determined by the radiation oncologist) must be presented at the peer review meeting before initial treatment. All cases were not discussed due to time and workload constraints. The ESTRO definition of reirradiation, namely “a new course of radiotherapy, either to a previously irradiated volume (irrespective of concerns of toxicity) or where the cumulative dose raises concerns of toxicity“ [11] was employed. Peer review meeting attendees include radiation oncologists, radiation therapists, radiation oncology medical physicists, and clinical fellows, with 5–10 participants attending the meetings. While meeting attendance changed weekly depending on staff availability, several radiation oncologists attended all meetings, and at least two radiation oncologists from each site, therapists and physicists were present at the meetings. The two sites were linked via video conference for the meeting and plans were presented by radiation therapists.

In each peer review meeting the treating radiation oncologist provided a brief review of the patient's history, including previous, concurrent, or planned chemotherapy and surgery, as well as the treatment intention. This was followed by a discussion of the treatment plan, including additional explanations or rationales for decision-making, and a review of any image fusions performed as relevant. Patient information, including radiological images, was available in electronic medical records and broadcast during discussions. This process is similar to the peer review process for patients undergoing treatment on the conventional linac, with the exception that MR simulation images are available for all MR-Linac patients to evaluate structures. Attendees were encouraged to ask questions and make suggestions during and after the case presentation regarding various aspects of the treatment plan, such as total dose and fractionation, target volumes, OAR volumes, margin expansion, and structure propagation. Changes were made through discussion and consensus agreement, with the radiation oncologist confirming the final changes (if any) and amending treatment plans accordingly. For a plan to be passed, the plan had to be approved by at least two non-treating radiation oncologists present at the peer review meeting. If there was a disagreement concerning decisions, decisions were made by majority.

All patients scheduled for treatment on the MR-Linac at both centres were included in the study unless they had opted out of their data being used for research. All patients signed a consent form agreeing to inclusion in any research project as part of their standard radiotherapy treatment consent. This study received ethics approval from the University of Notre Dame Australia Human Research Ethics Committee (reference number: 2023-050F), and data use was approved by GenesisCare as part of the GenesisCare Oncology Outcomes Protocol (reference number: 2022/ETH00247).

Data was gathered from 16 peer review meetings held between 8 June to 21 September 2023. These meetings discussed the treatment plans of 50 individual patients, with five patients being discussed in two separate meetings, resulting in a total of 55 cases. Each patient's case was graded prospectively using the 2019 RANZCR PRAT, which assesses the completeness of clinical record documentation and the degree of changes to the treatment plan following peer review [5]. For peer review assessment, this study used the PRAT's scoring system, which assigns the following grades: “A” (no change), “B” (minor change), “C” (major change) and N/A (not applicable) and is included at Appendix 1 and available together with explanatory documentation at https://www.ranzcr.com/component/edocman/radiation-oncology-peer-review-audit-tool-2013/download. Major and minor changes are respectively defined in the PRAT as follows:

Major changes are defined as a change requiring replanning and modifications to the current plan and/or having a foreseeable effect on treatment toxicity or cancer outcomes in the view of the peer-review physicians (e.g. unacceptable and avoidable cardiac dose in a breast tangent plan, change in treatment paradigm, alterations in contours that were needed to prevent a geographic miss, clinically significant changes in dose to part of a target volume or a significant change to a critical OAR)
Minor changes are defined as changes that are required but do not lead to significant replanning. These changes can also involve a change in policy affecting similar plans in the future, or smaller modifications to target volumes to enable better coverage or OAR sparing with the implication that the original contours would still have been clinically acceptable [5].

The PRAT scoring system was applied to the following aspects of the treatment plan: treatment modality, immobilisation/motion management, imaging modalities and fusion, total dose and fractionation, target/OAR volumes, fields, target volume dose coverage, normal tissue dosimetry and treatment verification. Cases were analysed to determine the rates of major and minor changes made to the treatment plan following peer review, and the peer review time for each case was recorded.

## Results

### Case characteristics

During the study period, 112 cases, comprising 95 individual patients, were considered for radiotherapy at both sites. Of these, 55 cases (49.1 %) and 50 patients (52.6 %) underwent peer review. All 55 cases presented were adult patients. Most cases (76.4 %) had oligometastatic/oligoprogressive disease, with 4.8 % receiving treatment at the primary site. The majority of cases were treated with SBRT (83.6 %). Reirradiation cases (i.e. in-field or prior radiotherapy near the treatment site) comprised 30.9 % of cases, with composite plans presented in these cases. The most common treatment sites reviewed were the liver (32.7 %,) followed by lymph nodes (29.1 %), the pancreas (12.7 %), and the adrenals and prostate (10.9 % each). Our SBRT cases involved prescriptions of 30–50 Gy in 3–5 fractions while our non-SBRT cases involved prescriptions of 50–74 Gy in 20–32 fractions. We aim for 95 % of the PTV to receive at least 100 % of the prescribed dose, while ensuring the maximum dose remains between 120 % and 150 % of the prescribed dose. In some cases, coverage may be compromised to adhere to OAR dose constraints. During treatment, continuous real time 2D cine MRI was used to assess tumour motion. To account for motion, including intrafractional respiratory motion, we used a non-gated technique with abdominal compression and an internal target volume (ITV) obtained from a 4D CT. Table 1 displays a summary of our case characteristics.

**Table 1:** Tumour characteristics, rate of treatment plan changes and time of presentation.

	No. of cases (n = 55)	No. of treatment plan changes	Mean time of presentation (minutes)
**Disease stage**			
Oligometastatic/Oligoprogressive	42 (76.4 %)	16 (38.1 %)	6 (range 2–15)
Non-oligometastatic/Oligoprogressive	13 (23.6 %)	4 (30.7 %)	8 (range 3–15)

**Treatment modality**			
SBRT	46 (83.6 %)	18 (39.1 %)	6 (range 2–15)
Non-SBRT	9 (16.4 %)	2 (22.2 %)	8 (range 5–15)

**Previous radiotherapy at/near site**			
No	38 (69.1 %)	13 (34.2 %)	6 (range 2–15)
Yes	17 (30.9 %)	7 (41.2 %)	8 (range 2–15)

**Site of treatment**			
Liver*^#^	18 (32.7 %)	6 (33.3 %)	5 (range 4–10)
Lymph nodes*^	16 (29.1 %)	7 (43.8 %)	7 (range 3–12)
Pancreas	7 (12.7 %)	0 (0 %)	7 (range 5–15)
Adrenal	6 (10.9 %)	4 (66.6 %)	5 (range 2–7)
Prostate^^^	6 (10.9 %)	2 (33.3 %)	7 (range 3–15)
Thorax ^#^	4 (7.3 %)	2 (50 %)	10 (range 3–15)
Spleen	2 (3.6 %)	1 (50 %)	8 (range 8–8)
Nasopharynx	1 (1.8 %)	1 (100 %)	15

* 3 cases received radiotherapy to the liver and lymph nodes.

^#^ 1 case received radiotherapy to the liver and thorax.

^1 case received radiotherapy to the prostate and lymph nodes.

### Peer review results

**Fig. 1:**
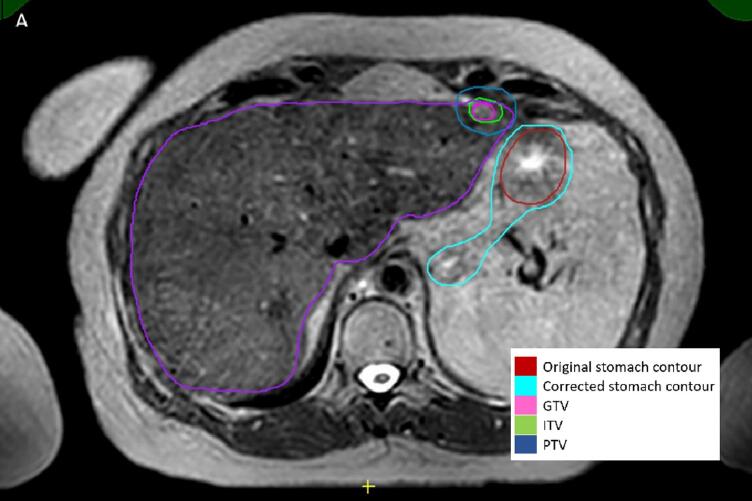
T2 navigated MRI scan of a patient with metastatic rectal adenocarcinoma with oligoprogressive segment 2 liver metastasis. Original stomach contour is denoted by dark red line. Pink, green and dark blue contours denote GTV, ITV and PTV. Turquoise line shows corrected stomach contour after peer review.

In this study, 36.4 % (n = 20) of cases experienced changes to the treatment plans following peer review meetings, with 3.6 % (n = 2) having a major change, where the decision was to defer treatment. In total, 24 changes were made across 20 treatment plans, necessitating replanning and reoptimisation before treatment commencement. A summary of post-peer review treatment plan changes is presented in Table 2. The most common changes were to OAR volumes (16.4 %, n = 9), followed by total dose and fractionation (10.9 %, n = 6) and target volume dose coverage (5.5 %, n = 3). Peer review resulted in changes to 39.1 % (n = 18) of SBRT cases and 41.2 % (n = 7) of reirradiation cases. For the five most common treatment sites, peer review resulted in changes to 33.3 % (n = 6) of liver cases, 43.8 % (n = 7) of lymph node cases, 66.6 % (n = 4) of adrenal cases and 33.3 % (n = 2) of prostate cases. Notably, no modifications were made to pancreas cases. An example of post-peer review change can be observed in Fig. 1, where initial OAR volumes were adjusted after peer review. The mean length of presentation was 7 min (range 2–15 min), with no major differences based on treatment modality, whether there had been previous radiotherapy, or the treatment site. Most cases took between 5 and 8 min to discuss, except for cases involving the thorax (10 min, 3 of which included mediastinal structures) and the nasopharynx (15 min).

**Table 2:** Changes made to treatment plans after peer review.

Any change	No. of changes	Mean time of presentation (minutes)
No	35 (63.6 %)	6 (range 2–15)
Yes	20 (36.4 %)	7 (range 3–15)

**Change categories**		
Minor	18 (32.7 %)	7 (range 3–15)
Major	2 (3.6 %)	12 (range 8–15)

**Type of change+**		
Treatment modality*	2 (3.6 %)	12 (range 8–15)
Immobilisation/motion management	1 (1.8 %)	8
Imaging modalities and fusion	1 (1.8 %)	9
Total dose and fractionation	6 (10.9 %)	7 (range 3–10)
Target volumes	1 (1.8 %)	8
Organ at risk volumes/dosimetry Organ at risk volume Organ at risk dosimetry	9 (16.4 %) 7 (12.7 %) 2 (3.6 %)	6 (range 3–15)
Fields	1 (1.8 %)	4
Target volume dose coverage	3 (5.5 %)	8 (range 5–15)

* One patient is on the ‘watch and wait’ list as the target was deemed too small to treat on MR-Linac and one patient was referred to receive brachytherapy.

## Discussion

This study evaluated the feasibility of incorporating pre-treatment peer review into the standard radiotherapy planning process for MR-Linac patients. It assessed the extent to which peer review resulted in changes to treatment plans, the effect of those changes, and time spent on peer review.

This prospective review of cases presented at weekly MR-Linac peer review meetings across two centres identified a major change rate of 3.6 % (n = 2), a total change rate of 36.4 % (n = 20) and a mean presentation time of 7 min. While there are no comparative studies evaluating peer review for MR-Linac patients, studies in conventional linac patients reported change rates ranging from 7.3 % to 47.8 %, with major change rates between 1.8 % and 27 % [2]. It is essential to acknowledge heterogeneity in outcome measurement methodologies across these studies, including quantification of post-peer review changes to treatment plans (e.g. some studies include changes that do not result in re-planning), the categorisation of changes as major/minor, and the parameters evaluated. For example, some studies categorise changes into dose, target or major changes that change treatment modalities [12], while others categorised changes by dose/fractionation, contouring, technique or a combination thereof [2]. Despite these variations, this study underscores a noteworthy rate of treatment plan changes, with a relatively low rate of major change. These findings highlight the significance of peer review, where minor yet detailed modifications can affect the treatment plans; without peer review, these changes would not have been made [1]. Fig. 1 demonstrates an example of a case of OAR modification after peer review.

This study also examined whether specific MR-Linac treatment indications (e.g. pancreatic cancer) required more emphasis in peer review on certain aspects of treatment planning. Notably, sites such as the liver, lymph nodes, adrenals and prostate had rates of change above 33 %, while no changes were made in pancreas cases. While we anticipated modest changes in pancreatic cases due to the proximity of OARs around the pancreas, difficulties in accurately visualising tumours due to poor soft tissue image delineation, and the associated risk of toxicity [4], our findings suggested otherwise. This finding could be attributable to various factors, including the relatively small size of our cohort, and the team's familiarity with pancreas cases.

Our study reported a reirradiation rate of 26.8 %, consistent with existing literature [12], [13]. The reirradiation cases in this study had higher rates of plan changes compared to de novo cases, in line with published data where reirradiation cases were found to be a major contributing factor to both OAR and prescription changes in the SBRT context [14]. Reirradiation with conventional radiotherapy has often been underused due to potentially severe long-term complications from normal tissue damage [15]. SBRT has been shown to be beneficial in reirradiation cases, due to its higher precision and tighter dose distribution allowing OAR sparing to mitigate toxicities [15], [16]. The majority of our cases involved SBRT (83.6 %), with 39.1 % of these treatment plans requiring changes following peer review, compared to non SBRT cases. In the context of conventional linac, SBRT cases were found to possess higher rates of change in treatment sites with low case volumes [14], highlighting that uncommon cases should undergo peer review. While SBRT is generally well-tolerated, real-time adaptation with MRgRT can reduce severe toxicity through better target dosimetry coverage while minimising dose to the OARs [4], [17]. It is essential to account for all possible sources of uncertainties and treatment planning challenges when prescribing SBRT. Coupled with the complexities associated with reirradiation, including patient selection, target volume definition, dose prescription and fractionation [15], this likely explains the increased rate of plan changes and discussion times. Our findings emphasise the important role of peer review, coupled with a robust planning process, in the management of SBRT and reirradiation cases on the MR-Linac.

Turning to the characteristics of post-review changes, the majority of plan changes in our study were to OAR volumes, followed by total dose and fractionation and target volume dose coverage. The high rates of OAR volume changes could be attributable to our newer centre being relatively inexperienced and less familiar with MRgRT initially, and we note no further OAR substructures were added on peer review. Changes were made to the OAR dosimetry for two patients due to the proximity of the OAR to the target, to ensure safe dose escalation to the target [18]. These findings are largely consistent with previous peer review studies, bearing in mind the abovementioned differences in how those studies measured outcomes. For example, a systematic review into peer review by Brunskill et al. observed that target volume delineation (45.2 %), dose prescription (24.4 %) and non-target volume delineation (7.5 %) were the most common reasons for the change in eight peer-review studies [19]. In contrast, only 1.8 % (n = 1) of our cases involved target volume delineation, possibly due to our sample size. Similarly, a 2019 peer-review study by Martin-Garcia et al. found contouring (targets and OARS) (53.8 %) and dose/fractionation (26.9 %) as the most common changes in their review of 148 cases [2]. In the SBRT context, Matuszak et al. conducted a study into pre-planning peer review for SBRT cases, whereby peer review of treatment plans including contouring was performed before weekly treatment planning meetings), and found that gross tumour volume and planning target volume were the most common changes [14].

As to timing, the mean presentation duration for most cases ranged from 5 to 8 min. Notably, thorax and nasopharynx cases required longer discussion times given that these indications are not commonly treated using MR-Linac in our centres. Cases involving major changes to treatment plans had longer mean presentation times than those involving minor changes (12 vs 7 min), which is unsurprising given the significance of a decision to defer treatment. None of the patients who had treatment deferred after peer review had subsequent radiotherapy during the study period. Consistent with previous peer review studies [1], [2], reirradiation cases also took marginally longer to discuss, likely due to the greater complexity of reirradiation cases. Finally, we did not specifically detect a learning curve over time but anticipate that with a longer observation period, there would be fewer changes over time, as observed by Ballo et al in their peer review study [12].

Peer review in radiation oncology is generally performed by public and private institutions across Australia and New Zealand, albeit with varying peer review processes across locations [20]. While time and resource limitations may preclude peer review in all cases, this study demonstrates the feasibility and benefits of implementing peer review in terms of changing treatment plans on the MR-Linac, highlighting the importance of this process for MR Linac departments. We note that in this study, approximately half of the cases at the two sites underwent peer review, with cases taking an average of 7 min to discuss. While all cases should be reviewed in an ideal world, this is unlikely to occur in any centre and is unlikely to be possible in a real-life clinical setting with limited time and resources [20], as well as other barriers such as increasing treatment complexity, planning resources per plan and cases involving complex medical decision-making regarding modality selection [21]. There is usually no opportunity to review all cases and contours in detail, though selected challenging cases may be discussed [22]. In addition, while the unique MR-Linac treatment workflow involving daily treatment adaptation theoretically permits daily peer-review, daily adapted plans were not peer-reviewed, and inter-fraction changes were not captured in our study. We do not consider peer-reviewing all fractions technically feasible due to OARs, target physiological migration during treatment adaptation, and resource limitations. However, we have previously reported on changes seen during treatment, including changes in dosimetry and OARs [23] and consider that reviewing patients between fractions would be valuable in allowing an assessment of what has been done, which would become the reference plan for the next fraction. Future work into post-peer review outcomes for MR-Linac patients at more treatment centres is necessary to identify the factors contributing to treatment plan changes, such as treatment site, modality and reirradiation. This could contribute to consensus recommendations for a standardised peer review process, including identifying cases requiring a greater focus or mandatory peer review.

In conclusion, this study allowed evaluation of the feasibility of the peer review process and the importance of it for treatment plans. Given the significant rate of treatment plan changes identified in this study, it is arguable that where possible, all MRgRT cases, especially those involving SBRT, oligometastatic/oligoprogressive sites, and/or reirradiation should undergo peer review, whether in-house or through combining different treating centres.

## CRediT authorship contribution statement

**Yew Sin:** Conceptualization, Data curation, Formal analysis, Investigation, Methodology, Project administration, Validation, Visualization, Writing – original draft, Writing – review & editing. **Vikneswary Batumalai:** Conceptualization, Data curation, Formal analysis, Investigation, Methodology, Project administration, Supervision, Validation, Visualization, Writing – review & editing. **Jeremy de Leon:** Resources, Writing – review & editing. **Eugene Leong:** Resources, Writing – review & editing. **Kasri Rahim:** Resources, Writing – review & editing. **Farshad Kasraei:** Resources, Writing – review & editing. **Charles Tran:** Resources, Writing – review & editing. **Tommy Liang:** Resources, Writing – review & editing. **Katrina Biggerstaff:** Resources, Writing – review & editing. **Michael G. Jameson:** Resources, Writing – review & editing. **Nicole Hug:** Resources, Writing – review & editing. **Kathryn Hird:** Conceptualization, Methodology, Project administration, Supervision, Writing – review & editing. **Hendrick Tan:** Conceptualization, Data curation, Formal analysis, Investigation, Methodology, Project administration, Supervision, Validation, Visualization, Writing – review & editing.

## Declaration of competing interest

The authors declare that they have no known competing financial interests or personal relationships that could have appeared to influence the work reported in this paper.

## Figures and Tables

**Table:** 

**Audit identifiers**		

Date of audit		
Patient ID		
Treating Radiation Oncologist		
Primary Site		
Date of starting RT		
Treatment intent	Curative/palliative/prophylactic/benign/not stated	
Y = Yes, N = No, N/A = Not applicable		

**Clinical record documentation**		

	**Complete**	**Comments**

Diagnosis		
Stage		
History and Examination		
Relevant Imaging		
Pathology		
Rationale for treatment		
Consent		
Letter or notes to referring doctor and/or GP		
A = No change, B = Minor change, C = Major change, N/A = Not applicable		

**Investigations and management**		

	Complete	Comments
Assessment/management		
Y = Yes, N = No, N/A = Not applicable		

**Other**		

	Status	Comments

Treated on protocol or practice guideline		
A = No change, B = Minor change, C = Major change, N/A = Not applicable		

**Radiation therapy plan review**		

	Change	Comments

Treatment modality		
Immobilisation/motion		
management		
Imaging modalities and fusion		
Total dose and fractionation		
Target volumes		
Organ at risk volumes		
Fields		
Target volume dose coverage		
Normal tissue dosimetry		
Treatment verification		
Y = Yes, N = No, N/A = Not applicable		

**Audit status**		

	Status	Comments

Date for next review		
Recommended changes completed		
Reasons why changes not implemented		
Review complete		
**Comments**		

## Appendix 1

RANZCR Peer Review Audit Tool 2019.
